# Increased ROS Production: A Component of the Longevity Equation in the Male Mygalomorph, *Brachypelma albopilosa*

**DOI:** 10.1371/journal.pone.0013104

**Published:** 2010-10-01

**Authors:** Francois Criscuolo, Candide Font-Sala, Frederic Bouillaud, Nicolas Poulin, Marie Trabalon

**Affiliations:** 1 Institut Pluridisciplinaire Hubert Curien, Département Ecologie, Physiologie et Ethologie, CNRS-UDS, UMR 7178, Strasbourg, France; 2 BIOTRAM, Université Paris Descartes, CNRS UPR9078, Faculté de Médecine Necker-Enfants Malades, Paris, France; University of Turku, Finland

## Abstract

**Background:**

The diversity of longevities encountered in wildlife is one of the most intriguing problems in biology. Evolutionary biologists have proposed different theories to explain how longevity variability may be driven by bad genes expression in late life or by gene pleiotropic effects. This reflexion has stimulated, in the last ten years, an active research on the proximal mechanisms that can shape lifespan. Reactive oxygen species (ROS), i.e., the by-products of oxidative metabolism, have emerged as the main proximate cause of ageing. Because ROS are mainly produced by the mitochondria, their production is linked to metabolic rate, and this may explain the differences in longevity between large and small species. However, their implication in the sex difference in longevity within a species has never been tested, despite the fact that these differences are widespread in the animal kingdom.

**Methodology/Principal Findings:**

Mitochondrial superoxide production of hemolymph immune cells and antioxidant and oxidative damages plasma levels were measured in adult male and female *B. albopilosa* at different ages. We found that female spiders are producing less mitochondrial superoxide, are better protected against oxidative attack and are then suffering less oxidative damages than males at adulthood.

**Conclusions/Significance:**

In tarantulas, once reaching sexual maturity, males have a life expectancy reduced to 1 to 2 years, while females can still live for 20 years, in spite of the fact that females continue to grow and moult. This study evidences an increased exposure of males to oxidative stress due to an increase in mitochondrial superoxide production and a decrease in hemolymph antioxidant defences. Such a phenomenon is likely to be part of the explanation for the sharp reduction of longevity accompanying male tarantula maturity. This opens several fundamental research roads in the future to better understand how reproduction and longevity are linked in an original ageing model.

## Introduction

The origin of the chelicerata phylum finds its roots at the precambrian ages and this long history has allowed extensive diversification of the chelicerate life history traits. Mygalomorphae are large, predatory spiders of the family *Theraphosidae* and adult sizes range from 4 to 10 inches across the outspread legs, spiders being able to catch prey size up to mice and birds [1]. *Theraphosidae* are renowned for their longevity. For example in the spiders of the subfamily *Theraphosinae*, the males can reach maturity in 4–8 years, living then only one – two year after the last molt (imaginal molt). In the same species, females reach maturity in 5–10 years and then live 20 more years while continuing to grow and resuming numerous reproductive cycles [2].

Sex differences in longevity are widespread in the animal kingdom and are indeed in general biased towards females [3], [4]. In a recent study, genetic modified mice having only female genes are living longer than control mice [5]. However, the mechanisms of sex differences in longevity remain to be determined and are likely to be species specific. For example, telomere length which is linked to longevity is longer in females than males in humans [6], [7]. Additionally, telomere loss after an infection is greater in males than in females [8]. Because telomere loss is mainly triggered by oxidative stress [9], differences in oxidative stress may be an important factor in determining female and male life expectancy [10], [11]. Therefore intra-specific differences in longevity would result from a more rapid senescence in males due to a “live fast, die young” trade-off, which can be traduced, for example, by higher chronic oxidative stress [12], [13].

Mygalomorphes are an ideal model to tackle sex differences in life history strategies, and to look at the intimate physiological mechanisms that uphold the different trade-offs. Once reaching sexual maturity, both sexes largely differ in their living modes. Males adopt a very active life notably related to the active search for reproductive partners [14] which induces higher energetic demands and leads to a higher resting metabolic rate (RMR) compared to females [15]. Based on the “free radical theory of ageing”, higher metabolic rate should induce higher ROS production and without the appropriate antioxidant protective answer, high RMR will be associated with increased senescence rates [16]. However, counter-intuitive results have been found at the individual level in mice, where high RMR are associated with longer lifespan because of specific mitochondrial adaptations [17], [18]. It remains that males generally show at the end of the reproduction period the resulting consequences of a higher rate of ageing, by losing their abdomen hairs, losing body mass and an overall deterioration of body condition and of locomotor abilities [19].

In the present paper, we tested in captive tarantula Theraphosidae (*Brachypelma albopilosa*) whether sexual maturity is accompanied by an increase in oxidative stress in males. To assess oxidative stress, we measured superoxide mitochondrial production in haemocyte cells and the ratio antioxidant defences/oxidative damages in the hemolymph. Based on the free-radical hypothesis of ageing, we predict that sexually mature males should have an enhanced production of mitochondrial ROS associated with a less efficient antioxidant barrier, increasing their chronic level of oxidative damages.

## Results

Description of life trajectories of captive tarantulas is given in Table 1, confirming that in controlled conditions, females lived longer than males (data presented in Table 1 were obtained from a different set of individuals). Additionally, there was a significant difference in body mass between sexes, males *B. albopilosa* being nearly 3 fold lighter than females (10.84±0.78 *vs.* 28.88±0.80 g, F_1, 28_ = 260.7, *P*<0.001).

**Table 1:** Longevity of *Brachypelma albopilosa* in laboratory (n = 30 for males and n = 10 for females).

	Males	Females
**Duration of development**	Days	Days
**Emergence from egg sac to adult moult (maturity)**	1792±22	2034±29
**Adult period: adult - death**	437±105	5790±712
**Total longevity**	2229±127	7824±741

These data were measured from different individuals than the ones used for superoxide production. The duration of periods were compared between males and females using a *t*-test. All inter-sex comparisons are significant, *P*<0.001.

### Antioxidant capacity and level of oxidative damage

**Figure 1:**
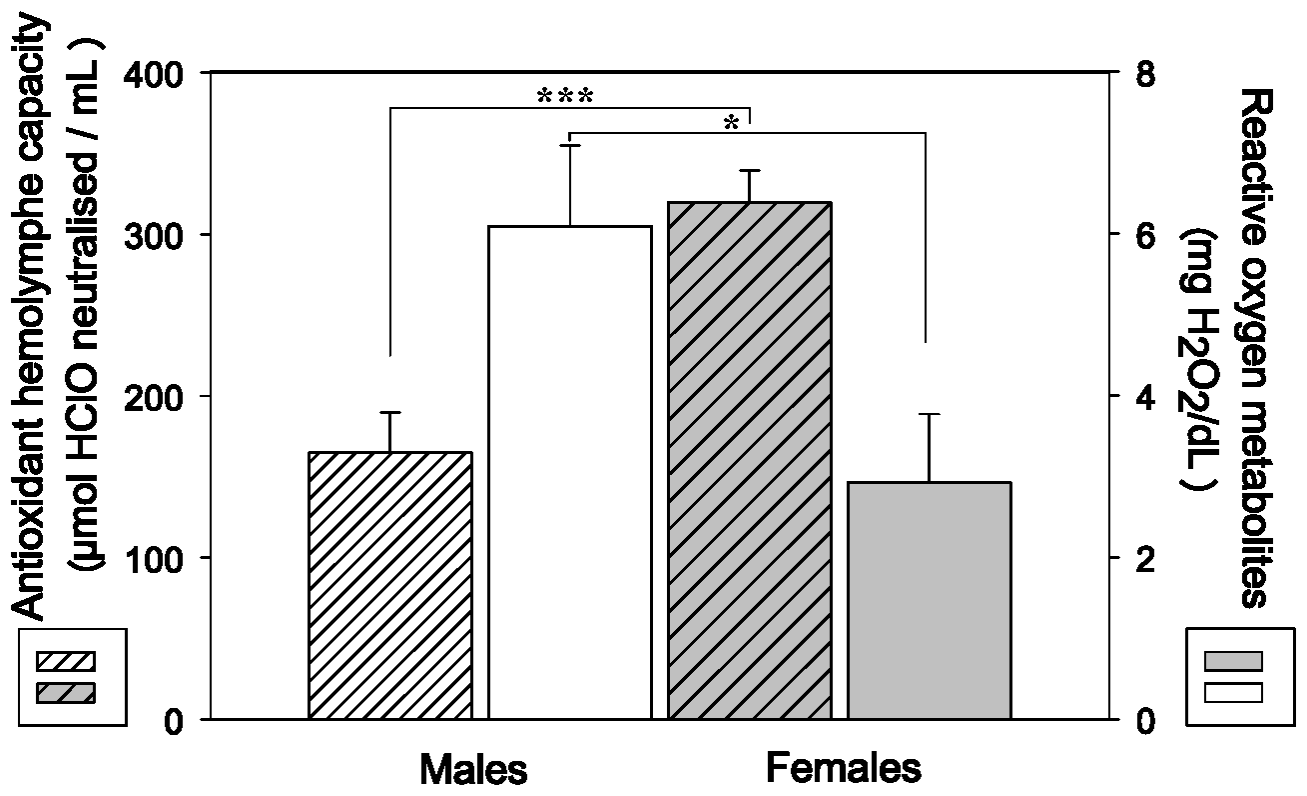
Antioxidant hemolymph barrier measured on male (white cross hatched bars, n = 9) and female (grey cross hatched bars, n = 11) tarantulas, as well as reactive oxygen metabolite levels (5 males (white bars) and 7 females (grey bars)). Female tarantulas showed a two fold higher antioxidant capacity than males (*P*<0.001), and a two fold reduced quantity of damaged bio-molecules in their haemolymph (*P* = 0.036).

Fig. 1 shows the antioxidant capacities and the hydroperoxide levels found in male and female tarantula hemolymph. Antioxidant barrier was significantly higher in females (382.3±18.7 *vs.* 169.3±20.6 mM of HClO neutralised, F_1, 19_ = 58.55, *P*<0.001). Conversely, the concentration of hydroperoxides was higher in males (6.09±1.00 *vs.* 2.92±0.85 mM H_2_O_2_ neutralised, F_1, 11_ = 5.841, *P* = 0.036), thereby showing that, on average, females have a more efficient antioxidant capacity and accumulated less oxidative damages.

### Cell superoxide measurement

**Figure 2:**
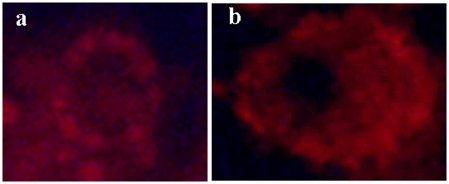
Representative images of tarantula haemolymph cells after incubation with MitoSOX Red probe. Fluorescence was mainly localized in the cytoplasm (a) and there was no apparent unspecific labelling of the nucleus (b).

**Figure 3:**
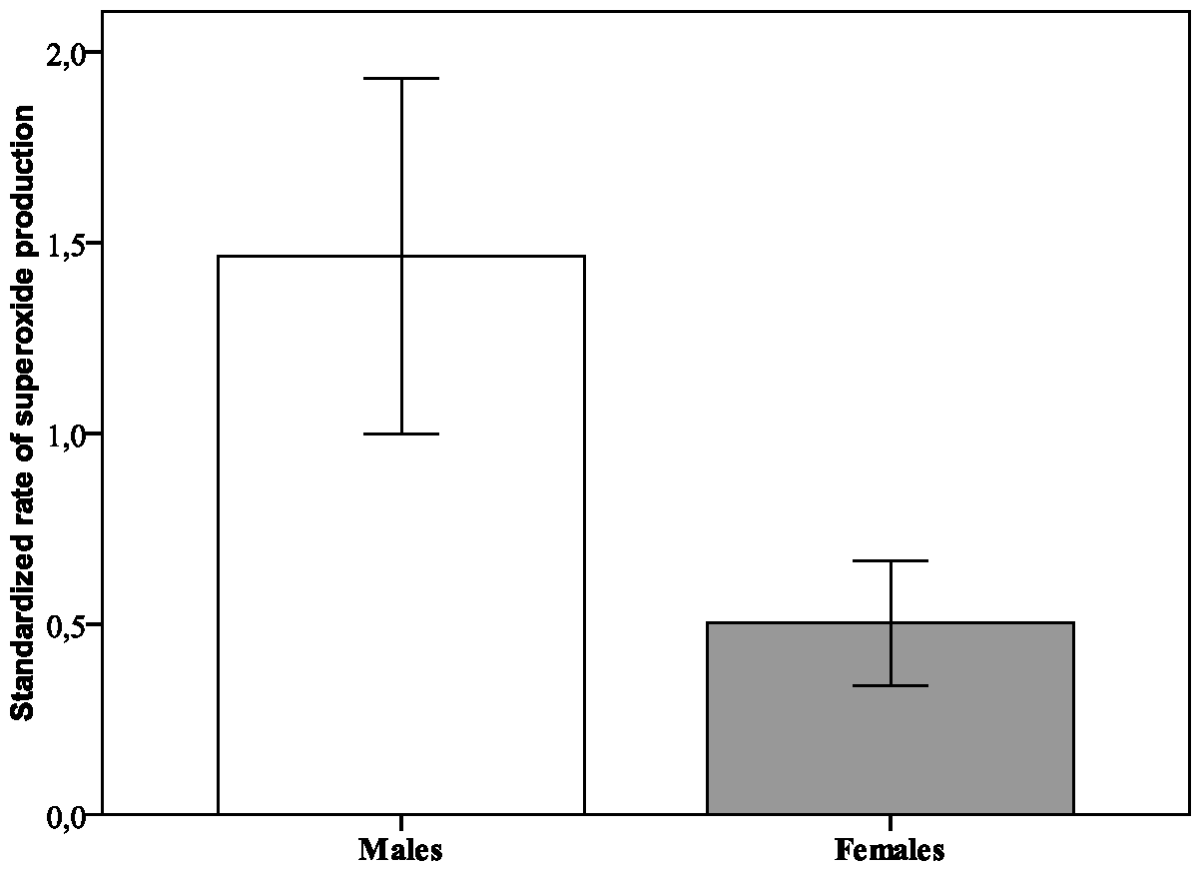
Mean superoxide production measured using MitoSOX Red fluorescence intensity levels in haemolymph tarantula cells. Data were collected during three independent sessions. Rate of superoxide production were standardized among the three sessions of measurement and examined relatively to each mean values. Males (n = 15) presented a relative superoxide production significantly higher than females (n = 14, Table 1).

MitoSOX fluorescence was not found in the nucleus (a noticeable drawback), thereby confirming the specificity of the probe as a superoxide indicator (Fig. 2). The rate of oxidation of MitoSOX™ Red probe in haemolymph cell determined in *B. albopilosa* was significantly different between sexes (Linear Mixed Model, F_1, 26.71_ = 19.14, P<0.001). Males exhibited a higher mean rate of superoxide accumulation compared to females (1.47±0.18 *vs.* 0.50±0.18 slope value, Fig. 3) and this was independent of any session effect (estimated covariance parameter: 0.08±0.13).

**Figure 4:**
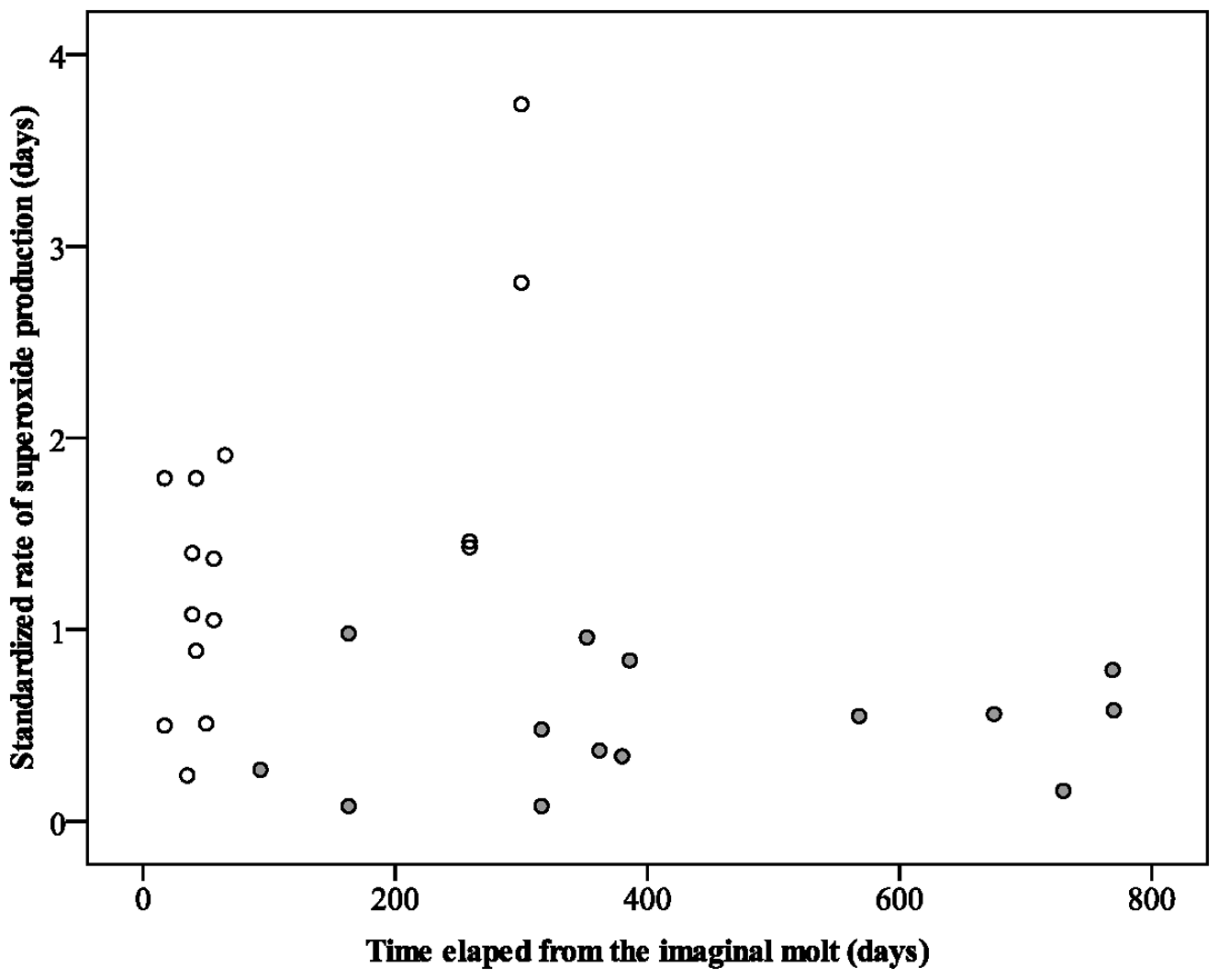
Standardized superoxide production in 15 males and 14 females tarantula, measured in haemolymph cells, in relation to the time elapsed from the imaginal moult. Each point represents the rate of accumulation of superoxide during two hours measured using MitoSOX fluorescence in one individual (see text for statistics).

Two different factors may account for this difference in superoxide production: body mass, and the time elapsed from the imaginal moult because of the sex differences in adult lifespan and development. We checked for within sex-effect (Table 2) and we found that: (i) superoxide production was not affected either by the time or by the body mass residuals in females and (ii) superoxide production increased with time when male tarantulas became adult, independently of body mass variability with time (Fig. 4).

**Table 2:** General linear model testing the effect of two factors, the time elapsed from the last moult and the residuals between body mass and the time elapsed from the last moult on mitochondrial ROS production in males or females tarantula.

	df	F	*P*
**Cellsuperoxideproduction**			
**Males**			
**Time from last moult**	1,14	11.20	**0.005**
**Residuals Body mass/Time from last moult**	1,14	2.96	0.111
**Females**			
**Time from last moult**	1,13	0.22	0.645
**Residuals Body mass/Time from last moult**	1,13	1.50	0.244

Linear regression values for males were: ROS  = 0.006 x Time from last moult +0.88, r = 0.67. Confidence interval at 95% for the slope value: 0.002–0.009; for the constant: 0.341–1.417.

## Discussion

Male tarantulas are exposed to higher risk of oxidative stress. Rate of superoxide production measured on haemolymph cells revealed a significantly higher production in males. Moreover, a lower antioxidant capacity of the haemolymph was found in males. Therefore, reaching adulthood is synonymous of an unbalanced ROS production which can be one of the proximal mechanisms explaining the rapid ageing process observed in male tarantulas. Interestingly, adult females do not suffer from an increased oxidative stress with age, thereby suggesting that the roots of accelerated ageing in males are associated with, or originate from, the metabolic changes that accompanied male sexual maturity.

Two previous studies investigated physiological differences between male and female tarantulas and tried to established relationships between life history sexual dimorphism (males being active while females stayed sedentary in their burrows) and physiological changes [20], [15]. Because for some insects, reproductive success largely depends on male searching ability [21], it must have driven the selection of physiological traits that allow males to sustain intensive locomotor activity, such as a higher RMR. High RMR allows greater workload at peak times [22] and it seems logical that basal metabolic rate has been found higher in male than in female spiders [15]. However, the same authors attested that peak metabolism is not submitted to sexual dimorphism, females being able to sustain the same maximal locomotor effort than males [20]. Because high RMR increases the overall energy demands of the body, one possibility is that males are not able to meet the overall energy requirement during reproduction, and are then facing an impossible trade-off, ending with the sacrifice of body maintenance. Data describing free-living male energy budget during reproduction should help to test this possibility. However, our growing conditions did not allow intense locomotor activity (cages being too small) and spiders were fed *ad libitum*. Males apparently continued to feed until reaching an advanced age (Trabalon, unpublished data), and despite this apparent balanced energy state, they were still presenting progressive body deterioration. This illustrates that male senescence rate at adulthood is not the mere consequence of a male lifestyle. Oxidative stress is the main mechanism put forward to explain the metabolic rate theory [16], [23], which stipulates that lifespan is inversely correlated to metabolic rate. Three important predictions derive from this metabolic rate theory of aging: (i) death probability increases with age, (ii) metabolic rate and lifespan should be inversely correlated to body size (large animals will have lower metabolism and longer life expectancy) and (iii) lifespan should be inversely correlated to ROS production and positively with antioxidant power. Our data give some supports to the two last predictions. However, the metabolic rate of living theory is faced with recent contradictions [24], [25], [26] and there is a need to further characterize how metabolic rate and oxidative stress can be or could be modified throughout the life stages in a longitudinal study of male and female tarantulas.

The metabolic shift from passive to active life may have co-evolved with a complete redistribution of reserves towards reproductive investment at the expense of body maintenance (the so-called cost of reproduction, [27]). However, the initial idea of functions competing for a limited amount of energy is still under debate [28] and energy expenditure associated to gamete production is supposed to be low in spider [20]. One possibility is that males are not trading-off a limited quantity of nutrients, but key metabolites among different functions. For example, male adult spiders use an amount of energy and metabolites in the spermatic web construction necessary for the transfer of sperm to copulatory organs [29]. Variability in internal allocation of metabolites has been previously shown in insects [30] and concerns cell fuels (lipids, glycogen) which may change the way mitochondria are functioning and finally drive different rates of ROS production [31]. Another possibility is that sexual maturation alters the nature of cell components (lipid membrane or proteins) in a way that their intrinsic resistance to oxidative stress is reduced in males [32]. Additionally, the active life style of adult male tarantulas exposed them to increased risks of mortality, either due to predation, sexual cannibalism, or to heat stress and desiccation since they abandon their burrows [14], [33]. Therefore, selection could have driven a lower investment in somatic maintenance in adult males and a greater allocation of metabolic resources to reproduction, leading to a reduced lifespan as predicted by the soma disposable theory [34].

Energy demanding sexual-related activities can alter immunity in vertebrates [35] as well as in invertebrates [36]. Most of the time, this cost is reflected as a diminution of immune efficiency, increasing the risk of infection [37], [38], [39] or of co-lateral autoimmune damages due to non-specific ROS production mainly by innate immune cells [40], [41]. In insects, haemocytes are circulating cells that are responsible for the cellular response by encapsulation and phagocytosis [42] during which ROS production will serve for pathogen destruction [43]. The available information on the immune system of spiders is scarce. However, ROS production during infection also occurs in arachnid haemocytes [44]. Therefore, because we mainly measured superoxide production in haemocytes, one question arises from our results: is it possible that male tarantulas suffer from autoimmune processes that may be involved in their fast ageing rate? It will be worthwhile to look at the importance of autoimmune disorders in old male *vs.* female tarantulas.

To conclude, our study suggests that oxidative stress is part of the proximal mechanisms explaining the great difference in longevity between male and female spider's *B. albopilosa*. Male reproductive strategy demands a sharp metabolic shift that is likely to be supported by changes in metabolism, which is apparently achieved at a cost for longevity. For example, juvenile hormone and insulin-like growth factors are known to affect longevity in insects [45], [46], [47], hypothetically by altering the immune system. An experimental approach manipulating this pathway or directly inhibiting the immune activation would help to better understand why male tarantulas present a semelparous reproductive strategy while females are iteroparous. As suggested in vertebrates [48], we confirmed that ROS production may be different between sexes at the intra-specific level, and that oxidative stress is likely to play a great role in shaping life history parameters.

## Materials and Methods

### Animal study

*Brachypelma albopilosa* [49] is a native of Costa Rica and Honduras terricolous spider. Listed on appendix II accordingly to the Convention on International Trade in Endangered Species (CITES), all spiders *B. albopilosa* used in the different tests came from the IPHC laboratory stock (permits N°540048 – Préfecture de Meurthe et Moselle, France). The spiders studied here emerged from five different egg sacs built by spiders in the laboratory. The spiderlings were reared and maintained until death. Twenty days after their emergence from the egg sac, the spiderlings were housed individually in 1 litter plastic containers (16×8 cm ×8 cm) during the larval period (1 year and four moult) and, from the four juvenile moults were maintained in 8 litters glass box (27×18 cm ×16 cm), before finally being transferred to 14 litters plastic containers (32×22 cm ×20 cm) after the pre-adult moult. All containers were lined with 2 cm of compost, which provided a substratum for locomotion, web fixation, refuge, attachment and ecdysis. The spiders were maintained under ambient conditions of temperature, humidity and luminosity. The air temperature and relative humidity were monitored daily using a hydro - thermograph. Breeding was induced at 25±2°C, under a 12∶12 h photoperiod and 60±10% of humidity. Spiders were fed *ad libitum* with a standardized diet of Tenebrionidae larvae (*Zophobas morio*) and of larvae or adults Blattidae (*Blabera fusca, Pleriplaneta americana*). These preys were selected according to the suitability of their body size for the experimental animals at different developmental stages.

Prior to sampling, spiders were anaesthetized by chilling at 4°C for 30 min and heamolymph was then sampled (0.5–1 ml) from punctured dorsal aorta at the edge between the cephalothorax and the opisthosoma into a capillary and flushed out into an Eppendorf tube kept on ice. Immediately after collection, haemolymph was diluted (1∶1) in anti-coagulant solution (NaCl 119 mM, NaHCO_3_ 14.9 mM, KCl 4.7 mM, KH_2_PO_4_ 1.18 mM, MgSO_4_ 1.17 mM, CaCL_2_ 1.6 mM, EDTA 0.026 mM, glucose 5.5 mM). Assessment of superoxide production was conducted immediately after haemolymph collection.

Superoxide measurement was conducted on 15 adult virgin males (1558.9±38.7 days) and 14 adult virgin females (3695.7±40.1 days), females being older than males (F_1, 28_ = 1471.8, *P*<0.001). Males and females tarantula have different moulting frequency. When the males reach sexual maturity and resume their imaginal moult, they stop growing and do not moult anymore [25]. On the contrary, once their imaginal moult is accomplished, females continue to grow and enter regular moulting periods through their adult life. In consequence, there was a great difference in the laps of time since the nympho-imaginal moult between sexes: males entered their reproductive life for 105.1±46.3 days while females did so for 431.6±47.9 days (F_1, 28_ = 24.06, *P*<0.001). In particular, if females have all reached sexual maturity since a long time, only four males were “old” mature spider (279.5±23.7 days since the imaginal moult) while eleven reached sexual maturity only for 41.6±15.2 days. Consequently, the post-moult period (days) was taken into account as a fixed factor in the data analysis (see Statistics).

### Antioxidant capacity and level of oxidative damage

Oxidative balance was assessed on frozen haemolymph from the same animals, depending on the volume of haemolymph remaining after the ROS production measurement. Consequently, the antioxidant barrier (in 11 females and 9 males *B. albopilosa*) and the concentration of reactive oxygen metabolites (primilary hydroperoxides, in 7 females and 5 males) were measured using OXY-Adsorbent and d-ROMs tests (Diacron, Italy). Detailed description of these measurements has been previously published [50], [51]. In Oxy test, the sample is subjected to massive oxidation through hypochlorous acid (HOCl), and the efficiency of its antioxidant capacity (including enzymatic and non-enzymatic compounds) is assessed by quantification of the unreacted (excess) of acid by a spectrophotometric method (λ = 492 nm). In D-ROM test, the concentration of cell hydroperoxides generated by oxidative attack on various organic substrates like proteins, lipids or DNA is measured, in presence of iron (produced by the R2 reagent), after generation of alkoxyl and peroxyl radicals which will in turn oxidize an alkyl-substituted aromatic amine (R1 reagent), thus transforming them into a pink derivate. Color development was quantified by spectrophotometry (λ = 492 nm), in proportion to the initial hydroperoxide concentration. Tarantula heamolymph was diluted 1/200 for linear determination using a linear standard curve method. Intra-specific (between male and female tarantulas, dilution in distilled water 1∶200) differences in the antioxidant capacity was determined. All measurements were run in duplicates. Intra-individual coefficient of variation was 4.9% for the Oxy test and 8.3% for the D-ROM test.

### Cell superoxide measurement

Mitochondrial accumulation of superoxide was measured with MitoSOX™ Red (Invitrogen), a highly selective fluorescent probe for the detection of ROS generated within the mitochondria. MitoSOX™ Red was added to the diluted hemolymph at a final concentration of 5 µM in the final media and incubated at ambient temperature (20°C) for 2 hours (time course of the measurement). Fluorescence intensity was recorded immediately after the addition of the probe (t0), and then during a kinetic measurement after 60 min (t60) and 2 hours of incubation (t120). Fluorescent signal emitted from the oxidized MitoSOX™ Red reagent was detected by flow cytometry (Epics Coulter XL4, Beckman-Coulter instrument, System II acquisition software). Rate of superoxide production during the kinetic measurement was quantified by plotting the fluorescent values with time (0, 60, 120 min) and by calculating the slope of the fit curve [52]. This slope value was used for statistical analysis. Measurements have been conducted in three different sessions and to control for inter-session variability in fluorescent data, all individual values have been standardized using the corresponding mean superoxide production value (of all samples measured in the same session). Values used for the statistical analysis are then expressed in relation to the mean session value (mean value  = 1). Superoxide production in both sexes has been measured for each session. MitoSOX superoxide measurement was repeated the same day for 4 individuals allowing the estimation of intra-individual coefficient of variation (9.3%). MitoSOX fluorescent signal may be affected by non-specific labelling (i.e. independent of the level of mitochondrial superoxide production) more specifically in the nucleus. Therefore, we checked for nuclear fluorescent signal by microscopy (Nikon Eclipse E600) and digital images of cells incubated with the fluorescent probe were taken using 60x oil immersion objective lens (digital camera DXM1200F).

### Statistics

Differences in antioxidant and oxidative damage levels were tested using a General Linear Model (GLM), with age and sex as fixed factors. Differences in superoxide production were tested using a Linear Mixed Model with sex used as a fixed factor and session number as a random factor since measures have been performed in 3 different sessions. Age, time from the imaginal moult and body mass were found to covary linearly, since animals get lighter with time. To avoid multilinearity, we chose to withdraw age from the analysis because (i) age of males and females were not overlapping and (ii) we were interested in adult differences in superoxide production. Consequently, time from the imaginal moult was preferred and used as a fixed factor. To discriminate the potential effect of body mass on superoxide production, we used the residuals of the relationship between body mass and time from the last moult, calculated separately for males and females because body masses did not overlap. These residuals gave us information on the effect of body mass on superoxide production independently of the body mass changes with time. Normality of the residuals was tested using Kolmogorov-Smirnov test. Analyses were performed using SPSS 16.0, with two-tailed tests and p values ≤0.05. Means are quoted ± S.E.
